# Mapping of Genetic Loci Conferring Resistance to Leaf Rust From Three Globally Resistant Durum Wheat Sources

**DOI:** 10.3389/fpls.2019.01247

**Published:** 2019-10-08

**Authors:** Dhouha Kthiri, Alexander Loladze, Amidou N’Diaye, Kirby T. Nilsen, Sean Walkowiak, Susanne Dreisigacker, Karim Ammar, Curtis J. Pozniak

**Affiliations:** ^1^Department of Plant Sciences, Crop Development Centre, University of Saskatchewan, Saskatoon, SK, Canada; ^2^International Maize and Wheat Improvement Center (CIMMYT), Mexico City, Mexico

**Keywords:** durum wheat, leaf rust, *Puccinia triticina*, resistance, quantitative trait loci, single nucleotide polymorphism (SNP)

## Abstract

Genetic resistance in the host plant is the most economical and environmentally friendly strategy for controlling wheat leaf rust, caused by *Puccinia triticina* Eriks. The durum wheat lines Gaza (Middle East), Arnacoris (France) and Saragolla (Italy) express high levels of resistance to the Mexican races of *P. triticina*. Three recombinant inbred line (RIL) populations, derived from crosses of each of these resistance sources to the susceptible line ATRED #2, were evaluated for leaf rust reactions at CIMMYT’s leaf rust nurseries in Mexico. Genetic analyses of host reactions suggested oligogenic control of resistance in all populations. The F_8_ RILs from each cross were genotyped using the Illumina iSelect 90K array, and high-density genetic maps were constructed for each population. Using composite interval mapping, a total of seven quantitative trait loci (QTL) that provide resistance to leaf rust were identified. Two QTL designated as *QLr.usw-6BS* and *QLr.usw-6BL* were identified on chromosome 6B in Gaza, which explained up to 78.5% and 21.3% of the observed leaf rust severity variance, respectively. A major QTL designated as *QLr.usw-7BL* was detected on the long arm of chromosome 7B in Arnacoris, which accounted for up to 65.9% of the disease severity variance. Arnacoris also carried a minor QTL on chromosome 1BL, designated as *QLr.usw-1BL.1* that explained up to 17.7% of the phenotypic variance. Three QTL conferred leaf rust resistance in Saragolla, namely *QLr.usw-2BS*, *QLr.usw-3B*, and *QLr.usw-1BL.2*, which accounted for up to 42.3, 9.4, and 7.1% of the phenotypic variance, respectively. Markers flanking each QTL were physically mapped against the durum wheat reference sequence and candidate genes involved in disease resistance were identified within the QTL intervals. The QTL identified in this study and their closely linked markers are useful resources for gene pyramiding and breeding for durable leaf rust resistance in durum wheat.

## Introduction

The importance of leaf rust as a threat to global durum wheat (*Triticum turgidum* L. ssp *durum*) production has increased dramatically during the last decade, due to the occurrence of highly virulent races of *Puccinia triticina* and the breakdown of the resistance genes that were widely deployed (Singh et al., 2004; Huerta-Espino et al., 2011; Herrera-Foessel et al., 2014a; Kolmer, 2015b; Soleiman et al., 2016; Kolmer and Hughes, 2017). Genetic control offers a cost-effective and environmentally friendly alternative to avoid yield losses due to this pathogen. Resistance to wheat leaf rust is commonly categorized into two classes based on their genetic control and phenotypic effect: race-specific, all-stage resistance, which is usually expressed as a hypersensitive response leading to host cell death, and adult plant resistance (APR), which is usually expressed as a slow-rusting phenotype (Knott, 1989; Lagudah, 2011; Kolmer, 2013; Singh et al., 2016). To date, over 77 leaf rust resistance (*Lr*) genes have been characterized and catalogued in wheat (McIntosh et al., 2017). Most *Lr* genes have major effects and confer race-specific, all-stage near-immunity. However, this class of resistance is prone to rapid breakdown as the pathogen population evolves, and new virulent races emerge (Suenaga et al., 2003; Huerta-Espino et al., 2011; Lowe et al., 2011; Ellis et al., 2014; Herrera-Foessel et al., 2014b). The over-reliance on a single race-specific resistance gene has led to leaf rust epidemics and considerable losses in the state of Sonora in Mexico, when the new fungal race BBG/BN overcame *Lr72* in 2001 (Singh et al., 2004; Herrera-Foessel et al., 2014a). Subsequently, loss of the resistance conferred by the complementary genes *Lr27*+*Lr31* occurred in 2008, with the emergence of race BBG/BP (Singh et al., 2004; Huerta-Espino et al., 2011). In contrast to race-specific resistance genes, APR genes express at the adult stage and only provide partial resistance which results in longer latent periods and smaller and fewer fungal spores or uredinia (Knott, 1989; Herrera-Foessel et al., 2008c; Lagudah, 2011; Lowe et al., 2011). Currently, at least eight genes that confer APR to leaf rust have been characterized in hexaploid wheat, including *Lr34* (Suenaga et al., 2003), *Lr46* (Singh et al., 1998), *Lr67* (Hiebert et al., 2010), *Lr68* (Herrera-Foessel et al., 2012), *Lr74* (McIntosh et al., 2015), *Lr75* (Singla et al., 2017), *Lr77* (Kolmer et al., 2018c), and *Lr78* (Kolmer et al., 2018a). Only *Lr46* has been reported in durum wheat (*T. turgidum* L. ssp. *durum*) (Herrera-Foessel et al., 2011).

Mapping quantitative trait loci (QTL) enables the detection of genes with both major and minor effects and the identification of linked molecular markers that could be used for gene stacking and breeding for durable rust resistance (Soriano and Royo, 2015). The advent of next generation sequencing technologies and high-throughput SNP genotyping platforms facilitated the development of high density genetic maps in wheat (Wang et al., 2014; Maccaferri et al., 2015; Winfield et al., 2016), enhancing our ability to dissect economically important traits such as disease resistance. Several studies have used QTL mapping to identify and tag genomic regions involved in leaf rust resistance in hexaploid wheat (Schnurbusch et al., 2004; Rosewarne et al., 2008; Rosewarne et al., 2012; Buerstmayr et al., 2014; Kolmer, 2015a; Soriano and Royo, 2015), however, only a few studies have been conducted to map QTL for leaf rust resistance in durum wheat (Marone et al., 2009; Singh et al., 2013a; Lan et al., 2017). The objective of this study was to use the iSelect 90K SNP array to characterize and map genetic loci conferring leaf rust resistance in three globally resistant durum wheat genotypes and to identify linked SNP markers useful for gene pyramiding and marker-assisted breeding.

## Materials and Methods

### Plant Materials and Field Phenotyping

Three sources of resistance to leaf rust (*P. triticina*), including the Middle Eastern landrace Gaza (unknown pedigree, CIMMYT genotype ID 233), the French cultivar Arnacoris (unpublished pedigree, CIMMYT genotype ID 6048080), and the Italian cultivar Saragolla (pedigree: Iride/Linea PSB 0114, CIMMYT genotype ID 255301), were identified by CIMMYT’s durum wheat breeding program, through extensive multi-race, multisite testing (data not shown). Each source was crossed to the susceptible line ATRED #2 (pedigree: Atil*2/LocalRed, CIMMYT genotype ID 5460557), and RIL populations of over 200 RILs each were developed by advancing generations through single-seed-descent, at the two locations used by CIMMYT’s breeding program: CENEB experimental station near Ciudad Obregon in Sonora (latitude 27.33, longitude -109.93, altitude 35 meters above sea level (masl)), with a wheat crop season from mid-November to late April, and El Batán experimental station, northeast of Mexico City (latitude 19.53, longitude -98.84, altitude 2250 masl), with a wheat crop season from mid-May to mid-October. Reactions to the widely virulent race of *P. triticina* BBG/BP (Huerta-Espino et al., 2011; Loladze et al., 2014) were scored on the F_2_ progenies during the spring of 2011 (CENEB). During summer 2011, the F_2_-derived F_3_ families (F_2:3_) were space planted in double 1.2-meter-long rows at El Batán. In 2013, F_6_ families were grown in replicated 1.2 m rows at the CENEB station, while paired 1.2 m rows of the F_8_ RILs were phenotyped at El Batán, in summer 2014. In all experiments, parental lines and progenies were inoculated at the tillering stage of plant development, using a mineral oil suspension of urediniospores of race BBG/BP of *P. triticina*, at a concentration of 5 to 10 mg of urediniospores per 5 ml of oil (Soltrol 170). Susceptible spreader rows surrounding plots and consisting of a mixture of the cultivars Banamichi C2004 and Jupare C2001 (susceptible only to race BBG/BP in Mexico) were also inoculated. The race BBG/BP of *P. triticina* is the predominant durum-specific race in Mexico, with the following avirulence/virulence formula: *Lr1*, *2a*, *2b*, *2c*, *3*, *3ka*, *3bg*, *9*, *13*, *14a*, *15*, *16*, *17*, *18*, *19*, *21*, *22a*, *24*, *25*, *26*, *28*, *29*, *30*, *32*, *35*, *37/Lr10*, *11*, *12*, *14b*, *20*, *23*, *27* + *31*, *33*, *72* (Huerta-Espino et al., 2008; Huerta-Espino et al., 2011; Loladze et al., 2014).

For the F_2:3_ families and the F_6_ RIL populations from the crosses Gaza/ATRED #2, Arnacoris/ATRED #2, and Saragolla/ATRED #2, as well as the F_8_ RILs from the Gaza/ATRED #2 cross, the modified Cobb scale was used to visually estimate the percentage of pustule-infected leaf area or leaf rust severity (LRS) on the parental lines and their progenies (Peterson et al., 1948). Three LRS scores were recorded at weekly intervals, starting at 14 days post inoculation, and the area under the disease progress curve (AUDPC) was calculated as before (Maccaferri et al., 2010). Host reactions were also recorded using four categories: “R” to indicate resistance or uredinia traces, “MR” to indicate moderate resistance with small uredinia surrounded by necrosis, “MS” to indicate moderate susceptibility expressed as chlorosis surrounding moderate sized uredinia, and “S” to indicate full susceptibility with large uredinia lacking necrosis or chlorosis (Roelfs et al., 1992). Based on the host reactions of plants within each family, the F_2:3_ families were categorized as homozygous resistant (R), homozygous susceptible (S), and heterozygous (Het). The F_8_ RILs from the crosses Arnacoris/ATRED #2 and Saragolla/ATRED #2 were scored as R or S. The chi-square (χ*^2^*) test was used to estimate the number of genes involved in the inheritance of leaf rust resistance in these populations. For analyses with a single degree of freedom, the chi-square values were adjusted with the Yates’s correction for continuity (Yates, 1934).

### Seedling Stage Evaluations of the F_2:3_ Families

Seedlings of F_2:3_ families from the crosses Gaza/ATRED #2, Arnacoris/ATRED #2, and Saragolla/ATRED #2 were evaluated for resistance to race BBG/BP of *P. triticina*, under controlled conditions in CIMMYT’s greenhouse, at El Batán experimental station. Approximately 25 to 35 seedlings from each F_2:3_ family were grown in 7-by-7-by-10 cm pots and inoculated with a suspension of urediniospores of race BBG/BP in light mineral oil (Loladze et al., 2014). Infection types (IT) were recorded using the 0–4 scale where “0” = no visible leaf rust symptoms; “;” = hypersensitive flecks without any uredinia; “1” = small uredinia surrounded by necrosis; “2” = small to medium uredinia surrounded by chlorosis or necrosis; “3” = medium-sized uredinia with or without chlorosis; “4” = large uredinia without chlorosis or necrosis; “X” = random distribution of variable-sized uredinia (mesothetic reaction), and “+” and “-” were used when uredinia were somewhat larger or smaller than the average for the IT class (McIntosh et al., 1995). ITs of 3, 3+, and 4 indicate susceptible host reactions, whereas all of the other ITs were considered resistant. Based on their ITs at 10 to 12 days after inoculation, the F_2:3_ families were classified as homozygous resistant “R,” homozygous susceptible “S,” or heterozygous “Het.” The χ*^2^* test was used to estimate the number of genes involved in the inheritance of leaf rust resistance at the seedling stage.

### Allelism Tests Between Gaza and Carriers of Known *Lr* Genes

Allelism between the resistance genes in Gaza and the known *Lr* genes *Lr61* and *Lr*_Camayo_ was investigated using 177 F_2_ plants from the cross Gaza/Sooty_9/Rascon_37//Guayacan INIA, and 273 F_2_ plants from the cross Gaza/Cirno C2008, respectively. Allelism to the *Lr* genes in the durum lines Geromtel_3 and Tunsyr_2 was also studied in populations of 326 and 181 F_2_ plants, respectively. The F_2_ progenies were tested for their field reaction to race BBG/BP of *P. triticina*, at El Batán. The resulting resistant/susceptible ratios were tested for goodness of fit to various gene models, using chi-square analysis. When no susceptible recombinants were detected in the F_2_ progenies, it was assumed that the two resistant parents carried allelic or closely linked leaf rust resistance genes.

### DNA Extraction and Illumina iSekect 90K SNP Array Genotyping

Genomic DNA was extracted from the parental lines and the F_8_ RILs from the three crosses Gaza/ATRED #2, Arnacoris/ATRED #2, and Saragolla/ATRED #2, using a BIOMEK FXp liquid handling station and the Sbeadex mini plant kit (LGC, Teddington, Middlesex, UK) (Dreisigacker et al., 2016). The Illumina iSelect 90K SNP array (Illumina, San Diego, CA, USA) was used for genotyping of the RILs and the parental lines (Wang et al., 2014). Genotype calling was performed using GenomeStudio software (Illumina, San Diego, CA, USA). The genotyping data for all three biparental populations are provided as **Supplementary Materials**. Prior to mapping, data filtering was carried out and markers that showed significant segregation distortion or more than 10% missing values were excluded.

### Construction of Linkage Maps and QTL Mapping

Curated SNP data were used to build linkage maps for each of the three populations. Initial linkage groups (LG) were obtained using the MSTMap software (Wu et al., 2008) with a stringent cutoff *p*-value of 1E^-10^ and a maximum distance between markers of 15.0 centimorgan (cM). The LGs were assigned to individual wheat chromosomes based on existing high-density SNP maps (Cavanagh et al., 2013; Wang et al., 2014; Maccaferri et al., 2015) and the tetraploid wheat reference sequences of wild emmer wheat (WEW) (Avni et al., 2017) and the modern durum wheat cultivar Svevo (Maccaferri et al., 2019). Once LGs were attributed to chromosomes, their genotypic data were pooled on a chromosome-by-chromosome basis and final genetic maps were constructed using MapDisto 1.7.7 software (Lorieux, 2012) at threshold values of recombination frequency = 0.3 and logarithm of the odds (LOD) = 3. Markers sharing the same segregation pattern (co-segregating) were identified in each LG, and the marker with the lowest percentage of missing data was chosen to represent each cluster or bin. Double recombinants were corrected using the functions “Show double recombinants,” “Show error candidates,” and “Replace error candidates by flanking genotype” as implemented in the MapDisto software (Lorieux, 2012). The order of the markers was determined using the Order, Ripple, and Check inversions commands. The Kosambi function was used to convert the recombination fractions to cM (Kosambi, 1943).

QTL detection was performed using the composite interval mapping (CIM) method implemented in QGene 4.4.0 software (Joehanes and Nelson, 2008). QTL were identified at a scan interval of 1 cM. The stepwise regression method was used to select cofactors and the LOD thresholds for determining statistically significant QTL were calculated by 1000 permutations with *P* < 0.05. The additive effect and percentage of phenotypic variance explained by each QTL were obtained from the final CIM results. Phenotypic traits analyzed included LRS, AUDPC and the host reaction (HR) recorded for the F_8_ RILs from the crosses Arnacoris/ATRED #2 and Saragolla/ATRED #2.

### QTL Interaction Tests in Each Population

A single marker with the highest LOD score was selected from each QTL to estimate its phenotypic effect. The mean phenotypic data for all the RILs from each population were grouped based on their genotype combinations at these selected loci and mean separation was performed using Fisher’s protected least significant difference (LSD) test in SAS 9.4 software (SAS Institute Inc., Cary, NC, USA).

### Genotyping of Molecular Markers Linked to Known *Lr* Genes

To test for presence of the APR gene *Lr46* (http://maswheat.ucdavis.edu/protocols/Lr46), the two simple sequence repeat (SSR) markers *Xwmc44* and *Xgwm259* (William et al., 2006) and a kompetitive allele specific polymerase chain reaction (KASP) marker were used to genotype the parental lines Arnacoris, Saragolla, and ATRED #2, as well as subsets of resistant and susceptible RILs from each population. The durum cultivars Kofa, CDC Verona, and Strongfield were used as checks. Three SSR markers (i.e. *Xwmc764*, *Xgwm210*, and *Xwmc661*) and two KASP markers (i.e. *kwm677* and *kwm744*) linked to the resistance gene *Lr16* (McCartney et al., 2005; Kassa et al., 2017) were used to genotype both parents and selected lines from the Saragolla/ATRED #2 population, as well as the check cultivar AC Domain. The parental lines Gaza and ATRED #2 and check cultivars including the *Lr61*-carrying Guyacan INIA were genotyped using the SSR marker *Xwmc487* linked to *Lr61* (Herrera-Foessel et al., 2008a). The four parental lines Gaza, Arnacoris, Saragolla, and ATRED #2 were also screened using the nucleotide-binding site leucine-rich repeat (NBS-LRR)-specific primers *4406F/4840R*, previously determined to be linked to *Lr14a* (Kthiri et al., 2018). The durum cultivars Sachem and Somayoa were used as positive controls that carry *Lr14a*. The amplification reactions were performed according to published protocols (McCartney et al., 2005; William et al., 2006; Herrera-Foessel et al., 2008a; Kthiri et al., 2018). Polymorphisms for the SSR markers and the NBS-LRR-specific primers were scored on 2% agarose gels.

### Physical Mapping to the Durum Wheat Reference Genome

The program GMAP (Wu and Watanabe, 2005) was used to align the sequences of the SNP markers that localized within each QTL LOD plot area to the genome sequence of the durum wheat cultivar “Svevo” (Maccaferri et al., 2019). Putative physical intervals for each QTL were identified using a cut-off value of 98% for sequence identity and sequence coverage. Genes that mapped within these physical intervals were identified using the available annotations for the durum wheat reference genome (Maccaferri et al., 2019).

## Results

### The Inheritance of Leaf Rust Resistance in Gaza, Arnacoris, and Saragolla

Leaf rust symptoms developed adequately for all the field evaluation trials at the CENEB and El Batán experimental stations. The RILs from the three mapping populations expressed a wide range in disease severity, with the resistant parents Gaza, Arnacoris, and Saragolla showing the lowest scores for leaf rust severity (0–5%), and the highest scores (90–100%) being observed on the susceptible parent ATRED #2. No transgressive segregation was observed among the RILs from the three crosses, which confirmed that the susceptible parent ATRED #2 does not contribute any genes for leaf rust resistance that could be detected under the present phenotyping conditions.

The F_2:3_ seedlings from the cross Gaza/ATRED #2 segregated at 63R:94Het:50S, which is a good fit to the 1:2:1 ratio expected for a single dominant seedling resistance gene, based on *p-*value (*P*_(< 0.05)_ = 0.18) of the χ*^2^* test at a 95% level of confidence (**Table 1**). However, field-based segregation ratio of 7R:8Het:1S (*P*_(< 0.05)_ = 0.12) of the F_3_ adult plants suggested the presence of two resistance genes in Gaza (**Table 1**). This discrepancy between seedlings and adult plants evaluation results led to the conclusion that Gaza could carry one APR and one seedling resistance gene. The involvement of an APR gene was further investigated by selecting 10 F_2:3_ families that were uniformly resistant in the field at the adult stage and uniformly susceptible at the seedling stage and testing them again at the adult plant stage, under greenhouse conditions. The results from these tests confirmed that the 10 selected families were indeed resistant at the adult stage. The F_8_ RILs from the cross Gaza/ATRED #2 showed a segregation of 1R:1S consistent with the segregation pattern of a single resistance gene (*P*_(< 0.05)_ = 0.65) (**Table 1**). This may be explained by the fact that APR genes are less effective in El Batán compared to the CENEB station (K. Ammar and A. Loladze, *unpublished*).

**Table 1:** Segregation ratios of the F_2:3_ and F_8_ progenies from three crosses of durum wheat lines evaluated for leaf rust resistance to race BBG/BP of *P. triticina*, at the seedling and adult plant stages.

Cross	Seedlings					Adult plants								
	F_2:3_ Families					F_2:3_ Families					F_8_ RILs			
	R	Het	S	Ratio	P	R	Het	S	Ratio	P	R	S	Ratio	*P*^a^
Gaza/ATRED #2	63	94	50	1:2:1	0.18	91	135	18	7:8:1	0.12	123	115	1:1	0.65
Arnacoris/ATRED #2	10	126	88	1:8:7	0.14	11	133	91	1:8:7	0.12	120	99	1:1	0.18
Saragolla/ATRED #2	59	102	46	1:2:1	0.43	9	95	104	1:8:7	0.15	82	122	1:1	0.01*
Saragolla/ATRED #2											82	122	3:1	0*

F_2:3_ families were categorized as resistant (R), heterozygous (Het), or susceptible (S). Mendelian ratios and their corresponding p-value (P) from chi-square (χ^2^) analysis are shown. The null hypothesis for the χ^2^ test was rejected at P < 0.05. * P < 0.05 indicating that the observed segregation ratio is significantly different from the expected segregation ratio at a 95% level of confidence. ^a^ The chi-square values were adjusted with the Yates’s correction for continuity.

Similar results were observed for the Saragolla/ATRED #2 population since segregation of the F_2:3_ seedlings fit a 1R:2Het:1S ratio (*P*_(< 0.05)_ = 0.43) expected for a single resistance gene, while the F_2:3_ adult plants segregation ratio fit a 1R:8Het:7S (*P*_(< 0.05)_ = 0.15) ratio, expected for the segregation of two resistance genes (**Table 1**). The screening of the F_8_ RILs, however, resulted in a distorted segregation of 82R:122S, which did not fit the expected ratios for Mendelian inheritance of one (*P*_(< 0.05)_ = 0.01) or two (*P*_(< 0.05)_ = 0) resistance genes (**Table 1**).

The 1R:8Het:7S segregation ratio observed for the F_2:3_ progenies from the cross Arnacoris/ATRED #2 at both the seedling (*P*_(< 0.05)_ = 0.14) and adult plant (*P*_(< 0.05)_ = 0.12) stages suggested the presence of two resistance genes in Arnacoris. However, the segregation ratio of 1R:1S (*P*_(< 0.05)_ = 0.18) observed in the F_8_ RILs was more consistent with that expected for a single resistance gene (**Table 1**).

**Figure 1:**
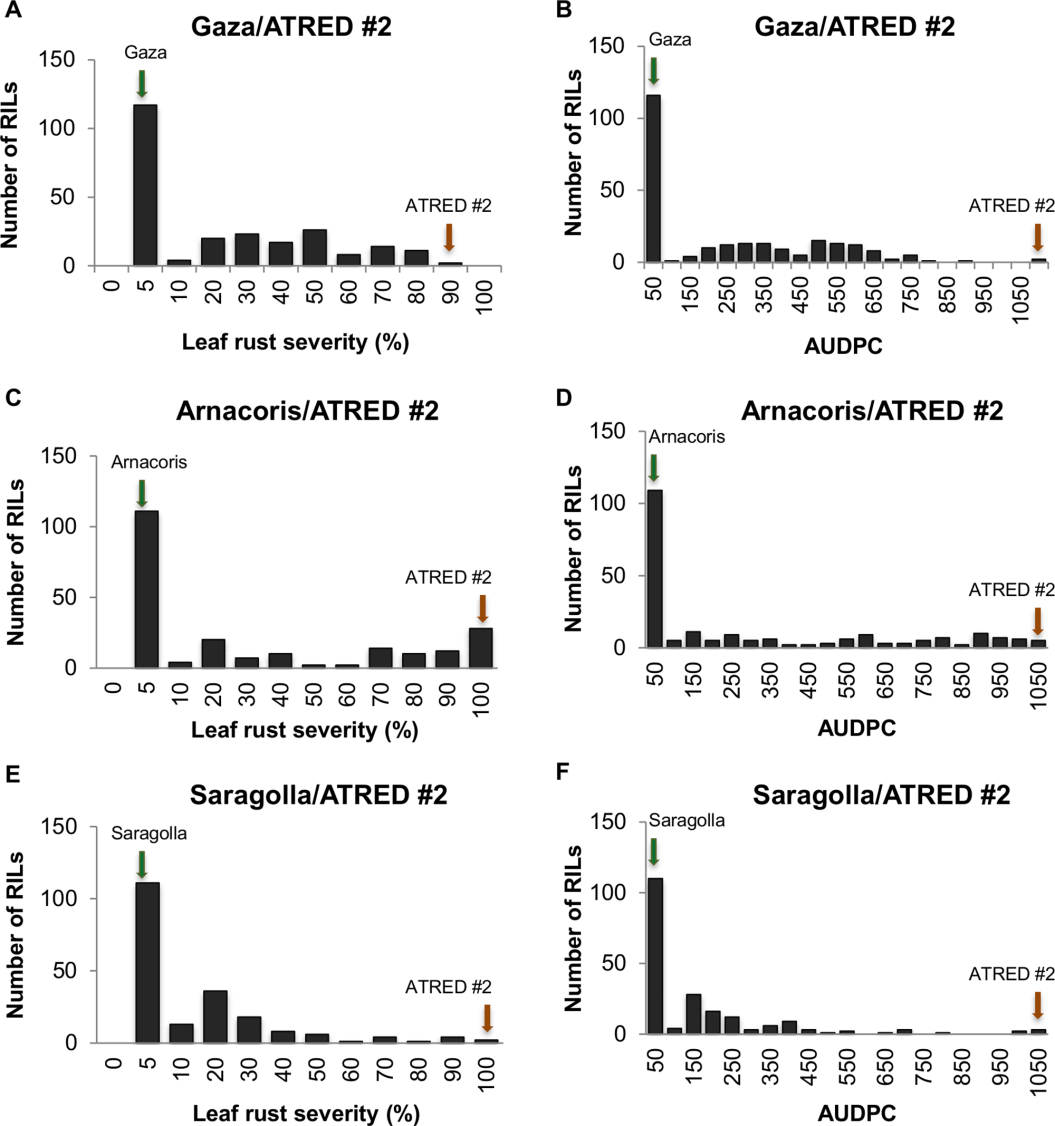
Frequency distributions of leaf rust severity and AUDPC in adult plants of the three RIL populations, evaluated for leaf rust reaction to the Mexican race BBG/BP in field plot tests. Panels represent **(A)** LRS for Gaza/ATRED #2, **(B)** AUDPC for Gaza/ATRED #2, **(C)** LRS for Arnacoris/ATRED #2, **(D)** AUDPC for Arnacoris/ATRED #2, **(E)** LRS for Saragolla/ATRED #2, **(F)** AUDPC for Saragolla/ATRED #2. Green arrows indicate positions of the resistant parents, and red arrows indicate positions of the susceptible parent ATRED #2.

The frequency distributions of the LRS and the AUDPC were determined for the three RIL populations (**Figure 1**). Although both disease severity and AUDPC data of the three populations showed continuous distributions, there was an obvious tendency of skewness towards resistance, which suggested that, while leaf rust resistance in Gaza, Arnacoris, and Saragolla was not monogenically inherited, there may be major gene effects in these populations.

### QTL Analyses Detect Several Leaf Rust Resistance Loci in Gaza, Arnacoris, and Saragolla

Polymorphic SNP markers with call frequencies ≥ 90% that fit the expected segregation ratio of 1:1 were considered reliable for mapping and were subsequently used to construct linkage maps for each of the three F_8_ RIL populations from the following crosses: Gaza/ATRED #2 (6248 SNP), Arnacoris/ATRED #2 (7315 SNP), and Saragolla/ATRED #2 (5345 SNP) (**Table 2**). The number of LGs ranged from 29 to 35, with all 14 durum chromosomes represented by at least one LG (**Table 2**). Comparison across genomes indicated that the maximum number of markers mapped to the B genome, except for Gaza where 51.2% of the SNPs mapped to the A genome. The high proportion of co-segregating SNP markers significantly reduced the final number of bins (unique marker loci) in the three maps. Marker density was greatest for the Arnacoris/ATRED #2 population, with an average inter-bin interval of 2.4 cM. However, the Gaza/ATRED #2 population produced the longest map with a total length of 4476.2 cM and the lowest marker density. The Saragolla/ATRED #2 population produced the shortest map with a total length of 3647.2 cM (**Table 2**). Final genetic maps for the three RIL populations are available as supplementary material (**Supplementary File S1**).

**Table 2:** Statistics of the three linkage maps from the three RIL populations Gaza/ATRED #2, Arnacoris/ATRED #2 and Saragolla/ATRED #2.

Resistant parent	Mapped SNPs	# of LGs	% A Genome	% B Genome	Map length (cM)	# of Bins	Average inter-bin distance (cM)
Gaza	6248	35	51.2	48.8	4476.2	1431	3.1
Arnacoris	7315	29	43	57	3745	1549	2.4
Saragolla	5345	33	35.8	64.2	3647.2	1293	2.8

#### QTL in the Gaza/ATRED #2 Population

Two QTL on chromosome 6B were associated with leaf rust resistance in the Gaza/ATRED #2 population. Both were derived from the resistant parent Gaza. The QTL were detected at a LOD significance threshold of 3.5, based on 1,000 permutation tests at a type I error rate of α < 0.05 (**Table 3**). The first QTL, *QLr.usw-6BS*, peaked at the locus *CAP7_c10772_156* and was detected in both F_6_ and F_8_ RILs, which were evaluated for leaf rust at CENEB in 2013 and at El Batán in 2014, respectively. This QTL was detected for both traits analyzed (LRS and AUDPC) at both locations, and explained up to 34.5% of the total phenotypic variance for AUDPC at CENEB (LOD 21.1) and up to 78.5% at El Batán (LOD 79.5) (**Table 3**). The second QTL detected in the Gaza/ATRED #2 progeny was designated as *QLr.usw-6BL* and peaked at the SNP marker *GENE-3689_293*. *QLr.usw-6BL* was also detected at both locations for all the traits analyzed, and accounted for 20.5% (LOD 11.4) and 18.7% (LOD 10.7) of the final LRS variance at CENEB and at El Batán, respectively (**Table 3**).

**Table 3:** Leaf rust resistance QTL detected by CIM in the Gaza/ATRED #2 population.

QTL	Flanking markers	Peak position (cM)	Trait	LOD	R^2^ (%)	Additive effect
QLr.usw-6BS	RAC875_c82406_177- CAP7_c10772_156	1.3	LRS_F_6_	15.5	26.7	-7.1
			AUDPC_F_6_	21.1	34.5	-94.3
			LRS_F_8_	70.7	74.5	-20.1
			AUDPC_F_8_	79.5	78.5	-189.6
QLr.usw-6BL	wsnp_Ex_c45713_51429315-GENE-3689_293	91	LRS_F_6_	11.4	20.5	-6.5
			AUDPC_F_6_	10.9	19.6	-69.3
			LRS_F_8_	10.7	18.7	-5.9
			AUDPC_F_8_	12.3	21.3	-53.1

**Figure 2:**
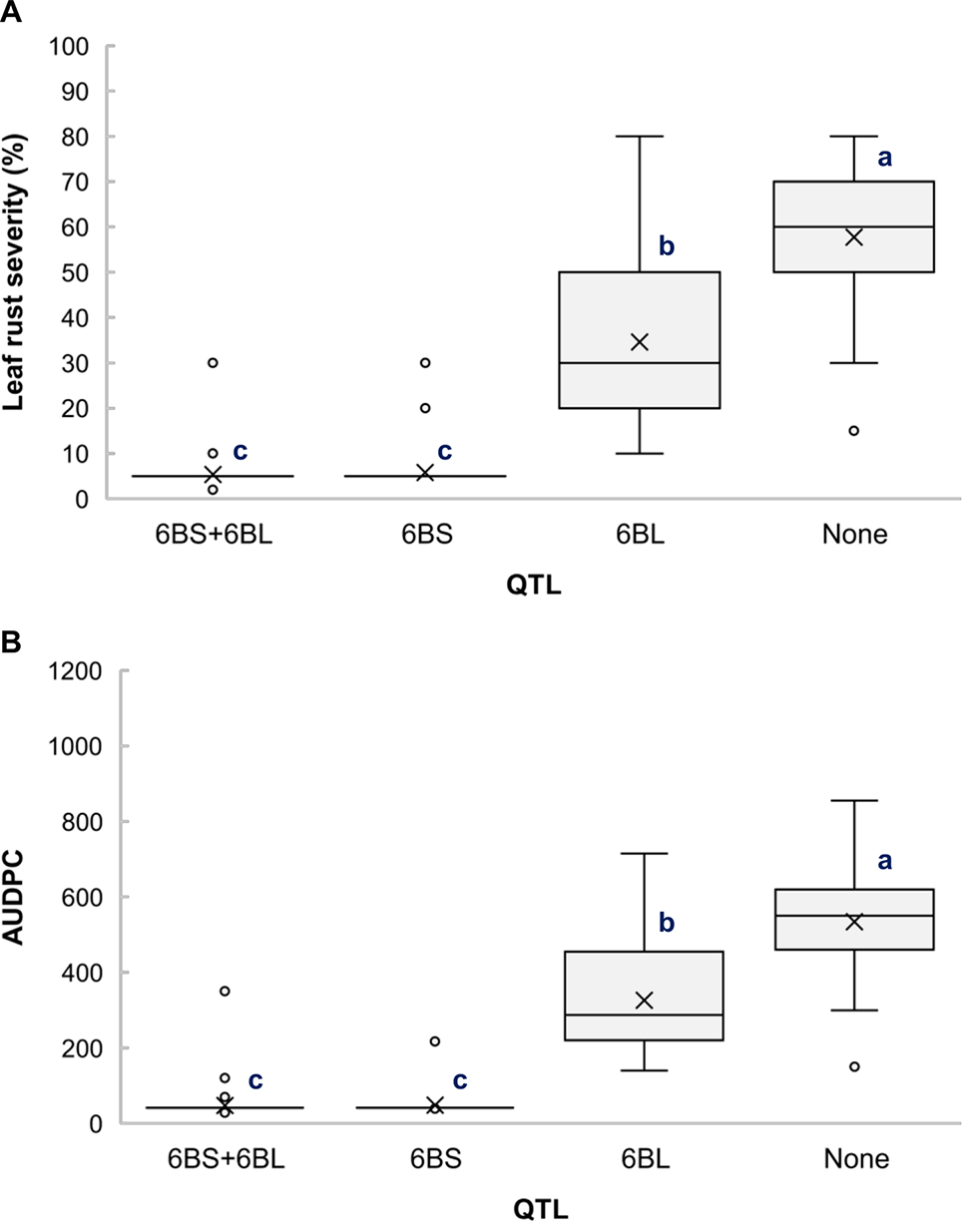
Boxplots illustrating the effects of *QLr.usw-6BS* and *QLr.usw-6BL* on **(A)** LRS and **(B)** AUDPC in the Gaza/ATRED #2 population. The QTL effects are shown in isolation and in combination. Means with the same letter are not signiﬁcantly different from one another at P < 0.05.

The RILs from the cross Gaza/ATRED #2 were grouped into four categories based on the allelic state of SNP markers *CAP7_c10772_156* and *GENE-3689_293* and mean separation was performed using Fisher’s LSD test. As expected, RILs that carried no resistance alleles scored the highest LRS and AUDPC of the allelic combinations (**Figures 2A, B**). *QLr.usw-6BS* had the strongest effect, since the mean LRS and AUDPC expressed by carriers of this QTL did not exceed 6% and 50.2, respectively (**Figures 2A, B**). *QLr.usw-6BL* was also able to reduce the disease symptoms, and though the reduction was not as strong as with *QLr.usw-6BS*, it was statistically significant. In general, the presence of *QLr.usw-6BS* reduced leaf rust symptoms to its lowest significant level, thereby masking the potential expression of *QLr.usw-6BL*.

The F_2_ progenies from the crosses Gaza/Sooty_9/Rascon_37//Guayacan INIA and Gaza/Cirno C2008 segregated at 55R:9S (*P*_(< 0.05)_ = 0.93) and 61R:3S (*P*_(< 0.05)_ = 0.44), respectively (**Supplementary Table S1**), indicating that the leaf rust resistance genes in Gaza are neither allelic nor linked to *Lr61* or *Lr_Camayo_*. The absence of susceptible recombinants in the F_2_ progeny from the cross Gaza/Geromtel_3 suggests that these two cultivars carry either allelic or closely linked *Lr* genes. However, the 61R:3S (*P*_(< 0.05)_ = 0.16) segregation ratio observed in the F_2_ population from the cross Gaza/Tunsyr_2 indicates that the *Lr* genes in these two cultivars are unrelated (**Supplementary Table S1**). The SSR marker *Xwmc487* linked to *Lr61* showed a clear difference in PCR product size between Gaza and the *Lr61*-carrying Guayacan INIA (**Supplementary Figure S1**).

#### QTL in the Arnacoris/ATRED #2 Population

Composite interval mapping revealed two QTL associated with resistance to leaf rust in the Arnacoris/ATRED #2 population (**Table 4**). A major QTL on the distal region of chromosome 7BL, designated as *QLr.usw-7BL*, with a peak LOD value at marker *BS00010355_51*, explained 65.9% of LRS variance (LOD 49.3) for the 2013 field trials at CENEB, and was highly significant for the AUDPC (LOD 46.1). *QLr.usw-7BL* was the only QTL detected for the host reactions of the F_8_ RILs evaluated at El Batán, and accounted for 99.6% of the phenotypic variance. A less significant QTL, designated as *QLr.usw-1BL.1*, was detected on the long arm of chromosome 1B for the 2013 field trials at CENEB (**Table 4**), explaining up to 17.7% of the phenotypic variance for the AUDPC (LOD 8.9) and *BS00060686_51* was the most significant marker within the interval.

**Table 4:** Leaf rust resistance QTL detected by CIM in the Arnacoris/ATRED #2 population.

QTL	Flanking markers	Peak position (cM)	Trait	LOD	R^2^ (%)	Additive effect
QLr.usw-1BL.1	BS00060686_51 - Kukri_c46030_412	0	LRS_F_6_	8.7	17.3	-8.9
			AUDPC_F_6_	8.9	17.7	-84.5
QLr.usw-7BL	Tdurum_contig30545_715 – Bobwhite_c42202_158	127	LRS_F_6_	49.3	65.9	-27.3
			AUDPC_F_6_	46.1	63.4	-239.3
			HR_F_8_	263.3	99.6	0.5

**Figure 3:**
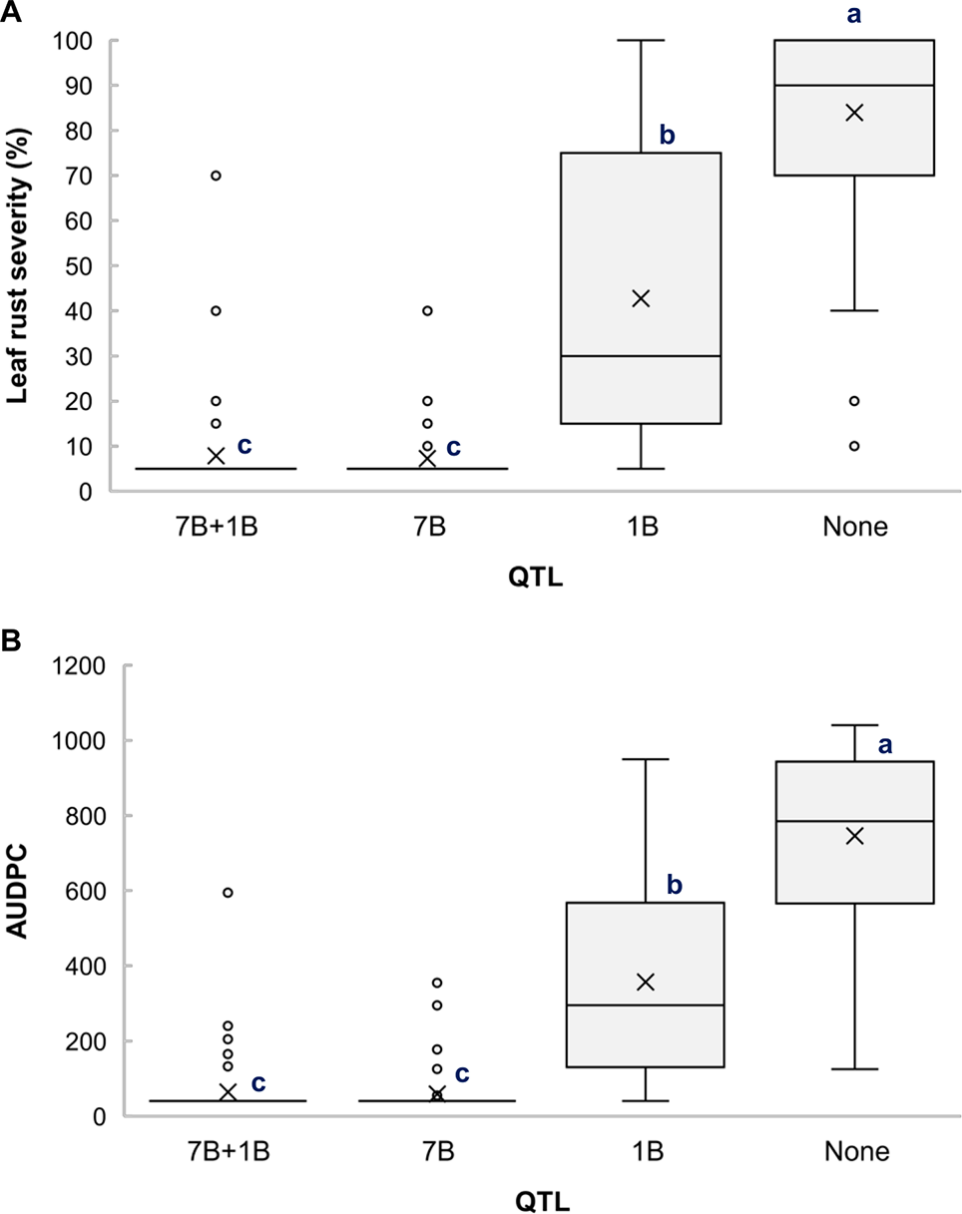
Boxplots illustrating the effects of *QLr.usw-7BL* and *QLr.usw-1BL.1* on **(A)** LRS and **(B)** AUDPC in the Arnacoris/ATRED #2 population. The QTL effects are shown in isolation and in combination. Means with the same letter are not signiﬁcantly different from one another at P < 0.05.

Based on the allelic state of the SNP markers *BS00010355_51* and *BS00060686_51*, the RILs from the cross Arnacoris/ATRED #2 were classified into carriers and non-carriers of *QLr.usw-7BL* and *QLr.usw-1BL.1*, and mean separation for LRS and AUDPC was performed using Fisher’s LSD test. Clearly, *QLr.usw-7BL* had the strongest effect on all the traits and its presence alone conferred the highest level of resistance (**Figures 3A, B**). The results also showed that *QLr.usw-1BL.1* was significant in reducing leaf rust symptoms only in the absence of *QLr.usw-7BL* (**Figures 3A, B**).

Molecular marker testing using the SSR markers *Xwmc44* and *Xgwm259* linked to the APR gene *Lr46* on chromosome 1BL showed inconclusive results, since both positive (Kofa) and negative (CDC Verona) controls had PCR products of similar size for *Xwmc44* (**Supplementary Figure S2A**), whereas marker *Xwmg259* had no PCR product in Arnacoris (**Supplementary Figure S2B**). However, genotyping with the KASP marker linked to *Lr46* showed that Arnacoris, as well as the RILs with and without *QLr.usw-1BL.1*, clustered with the susceptible parent ATRED #2 and the negative controls CDC Verona and Strongfield, suggesting that Arnacoris may not be carrying *Lr46* (**Supplementary Figure S3**). PCR amplification using the NBS-LRR primers *4406F/4840R*, which are linked to *Lr14a*, showed that the positive controls Somayoa and Sachem carry this marker, while Arnacoris had the null allele, which indicates that *QLr.usw-7BL* is different from *Lr14a* (**Supplementary Figure S4**).

#### QTL in the Saragolla/ATRED #2 Population

Three QTL controlled leaf rust resistance in the Saragolla/ATRED #2 population, which were detected by CIM on chromosomes 1BL, 2BS, and 3B (**Table 5**). *QLr.usw-2BS* peaked at 82 cM on chromosome 2B with *wsnp_Ex_c18354_27181086* being the most significant marker linked to this major QTL. *QLr.usw-2BS* explained up to 42.3% of the final leaf rust severity variance, and was the only QTL detected for the host reactions of the F_8_ RILs with a LOD score of 25.18. *QLr.usw-3B* peaked at marker *RAC875_rep_c82061_78* on chromosome 3B and accounted for 9.4% of the AUDPC variance. The minor QTL on the long arm of chromosome 1B, designated as *QLr.usw-1BL.2*, was flanked by markers *wsnp_Ex_c4436_7981188* and *BS00000010_51*, and explained up to 7.1% of the observed variance for AUDPC (**Table 5**).

**Table 5:** Leaf rust resistance QTL detected by CIM in the Saragolla/ATRED #2 population.

QTL	Flanking markers	Peak position (cM)	Trait	LOD	R^2^ (%)	Additive effect
QLr.usw-1BL.2	wsnp_Ex_c4436_7981188 - BS00000010_51	27	LRS_F_6_	2.02	4.6	-9.1
			AUDPC_F_6_	3.16	7.1	-109.8
QLr.usw-2BS	Tdurum_contig76118_145 - wsnp_Ex_c18354_27181086	82	LRS_F_6_	23.67	42.3	-12
			AUDPC_F_6_	20.95	38.6	-108.7
			HR_F_8_	25.18	44.3	0.3
QLr.usw-3B	Tdurum_contig33168_461 - RAC875_rep_c82061_78	13	LRS_F_6_	3.96	8.8	-4.1
			AUDPC_F_6_	4.24	9.4	-41.8

**Figure 4:**
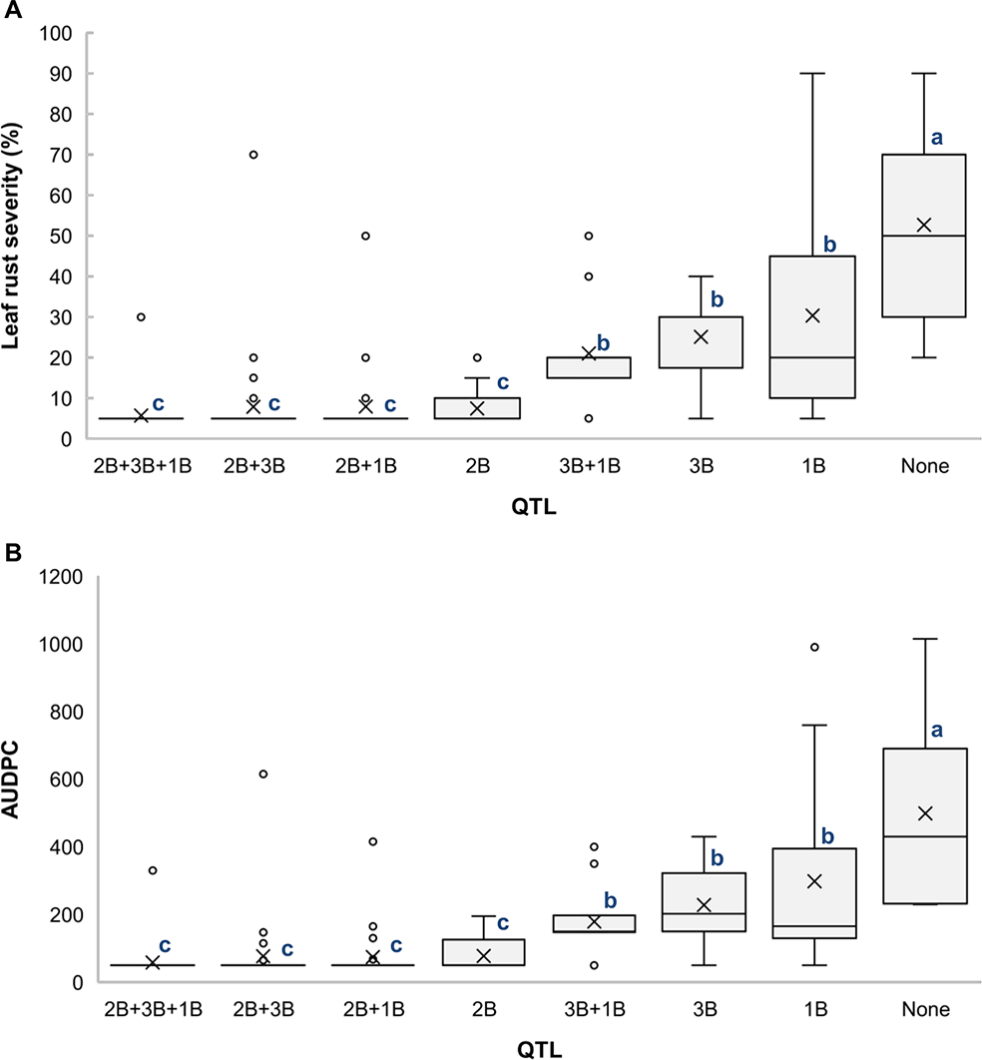
Boxplots illustrating the effects of *QLr.usw-2BS, QLr.usw-3B*, and *QLr.usw-1BL.2* on **(A)** LRS and **(B)** AUDPC in the Saragolla/ATRED #2 population. The QTL effects are shown in isolation and in combination. Means with the same letter are not signiﬁcantly different from one another at P < 0.05.

Analysis of different combinations of QTL based on the allelic state of SNP markers *BS00000010_51*, *wsnp_Ex_c18354_27181086*, and *RAC875_rep_c82061_78* showed that the RILs carrying all three QTL had the lowest averages for LRS (5.8%) (**Figure 4A**) and AUDPC (59) (**Figure 4B**), which indicates the additive effects of these QTL. It is also noticeable that *QLr.usw-2BS* had the major effect on leaf rust symptoms compared to both *QLr.usw-3B* and *QLr.usw-1BL.2*. However, both *QLr.usw-3B* and *QLr.usw-1BL.2* significantly reduced the leaf rust symptoms in the absence of *QLr.usw-2BS* (**Figure 4**).

Both parents and selected lines from the Saragolla/ATRED #2 population were genotyped with three SSR markers (*Xwmc764*, *Xgwm210*, and *Xwmc661*) linked to *Lr16* on chromosome 2BS (**Supplementary Figure S5**). The results showed polymorphism between the amplicons from Saragolla and AC Domain for the three markers, which suggests that *QLr.usw-2BS* is different from *Lr16*. The KASP markers *kwm677* (**Supplementary Figure S6A**) and *kwm744* (**Supplementary Figure S6B**) also showed polymorphism between Saragolla and the *Lr16*-carrying AC Domain.

### Physical Mapping to the Durum Wheat Reference Genome

The DNA sequences associated with the SNP markers mapping within each QTL LOD plot area in Gaza, Arnacoris, and Saragolla were physically mapped to the durum wheat reference genome of Svevo (Maccaferri et al., 2019) to identify candidate genes for leaf rust resistance (**Supplementary File S2**). Several genes identified within these QTL intervals encode proteins with motifs known to be associated with disease resistance such as NBS-LRR receptor proteins, calcium-dependant lipid-binding (CaLB domain) proteins, ATP-binding cassette (ABC) transporter proteins, as well as several receptor-like protein kinases (RLKs) (**Supplementary File S2**).

## Discussion

Diversification of the genetic basis of leaf rust resistance and breeding for durable resistance are both priorities in many durum wheat breeding programs worldwide, especially after the emergence of new virulent races of *P. triticina*. The goal of the present study was to characterize and map putatively uncharacterized genes for leaf rust resistance in the three durum genotypes: Gaza, Arnacoris, and Saragolla. Inheritance studies indicated the involvement of several loci that controlled leaf rust resistance in these lines, including at least one APR gene in each of Gaza and Saragolla. The segregation ratios of the F_2:3_ progenies from the Gaza/ATRED #2 population suggested one resistance gene in the seedlings evaluation and two in the adult plants evaluation. However, segregation of the F_8_ RILs adult plants suggested the presence of a single resistance gene. This discrepancy may be caused by the ineffectiveness of the Gaza APR gene in El Batán, since certain APR genes are known to be environment or temperature sensitive (Kaul and Shaner, 1989; McIntosh et al., 1995; Risk et al., 2012). For the Arnacoris/ATRED #2 population, the seedling and adult plant stage evaluations of the F_2:3_ progenies suggested the presence of two resistance genes, while the segregation ratio of the F_8_ RILs supported a single gene theory. Nonetheless, the F_2:3_ results were indeed supported by the QTL mapping results, since two QTL were detected in this population. The major phenotypic effect of *QLr.usw-7BL* suggests that this is an all-stage resistance gene, while *QLr.usw-1BL.1* is likely an APR gene, with a minor phenotypic effect. The discrepancy between the segregation ratios could be due to the differences between the climatic conditions during the years of evaluations. The F_2:3_ progenies were evaluated in El Batán in 2011, while the F_8_ RILs evaluation was conducted in 2014, and it is possible that the APR gene was not effective in 2014. The environmental effects on this gene warrant for follow-up field studies under various conditions. Although the material was inoculated with the race BBG/BP of *P. triticna* in both years, it is also possible that the racial constitution of the natural field population of the pathogen has been different between 2011 and 2014, thus affecting the results. The Saragolla/ATRED #2 population showed similar results for the F_2:3_ progenies, with the seedling data suggesting the presence of a single resistance gene, while the adult plant data suggested the presence of at least one additional APR gene. However, the segregation ratio from the F_8_ generation could not fit neither one nor two-gene models and we could not find a reasonable explanation for this aberration, which also calls for further evaluation of this cultivar under various environments.

Composite interval mapping identified genomic regions on chromosomes 1BL, 2BS, 3B, 6BS, 6BL, and 7BL, which were associated with leaf rust resistance in these three durum cultivars. These may be particularly valuable in breeding since they can be strategically combined to produce more durable resistance. The SNP markers linked to all the leaf rust resistance QTL identified in the present study were converted into KASP markers (**Supplementary Table S2**) and are currently being validated at CIMMYT.

### QTL on Chromosome 1B

The distal region of chromosome 1BL is known to carry the APR gene *Lr46*, identified in the wheat cultivar Pavon76 (Singh et al., 1998). The flanking SNP markers for *QLr.usw-1BL.1* in Arnacoris, *BS00060686_51*, and *Kukri_c46030_412*, mapped at 152.5 cM and 162.3 cM, respectively, on chromosome 1BL in the SNP-based consensus map of tetraploid wheat (Maccaferri et al., 2015). Likewise, the SNP marker *wsnp_Ex_c4436_7981188* that flanks *QLr.usw-1BL.2* in Saragolla, mapped at 145.4 cM on 1BL. The two SSR markers linked to *Lr46*, *Xwmc44* and *Xgwm259* (William et al., 2006), mapped at 140.6 and 150.6 cM, respectively, in the same durum consensus map (Maccaferri et al., 2015). Despite the proximity between these marker intervals, further molecular marker analyses showed that Arnacoris may not be carrying *Lr46* (**Supplementary Figure S3**). This is supported by the observation that *QLr.usw-1BL.1* alone has a much stronger phenotypic effect on disease symptoms reduction (**Figure 3**) compared to *Lr46*, when present alone in durum wheat (K. Ammar, *unpublished*). However, *Qlr.usw-1BL.2* present in Saragolla is likely *Lr46*, which is consistent with the observation that *QLr.usw-1BL.2* has a very moderate effect on disease symptoms reduction (**Figure 4**).

Other leaf rust resistance genes that map to chromosome 1B include *Lr33* (Dyck et al., 1987), *Lr44* (Dyck and Sykes, 1994), *Lr71* (Singh et al., 2013b), and *Lr26* (Mago et al., 2002; Mago et al., 2005). However, virulence in durum specific races is common for *Lr33*, such as the Mexican race BBG/BP that is used in the present study (Herrera-Foessel et al., 2008b; Huerta-Espino et al., 2011; Lan et al., 2017). Genes *Lr44* and *Lr71* were both identified in spelt wheat (*T. spelta*) and no reports indicate their presence in durum wheat. Furthermore, the SSR markers *Xgwm18* and *Xbarc187*, which are closely linked to *Lr71* (Singh et al., 2013b), mapped at 35.6 and 35.7 cM in the tetraploid consensus map, respectively (Maccaferri et al., 2015). Based on the position of the SNP markers flanking *QLr.usw-1BL.1* in the same consensus map at 152.5 and 162.3 cM, it is possible to conclude that Arnacoris does not carry *Lr71*. The short arm of chromosome 1R of rye (*Secale cereale*) carries the leaf rust resistance gene *Lr26* and has been widely used in wheat breeding programs through the 1BL.1RS (wheat-rye) translocation (Mago et al., 2005). However, there is no indication of the presence of the 1BL.1RS translocation in Arnacoris. While it is highly unlikely that *QLr.usw-1BL.1* harbor any of the three designated genes on chromosome 1B, definitive proof could not be obtained in the present study.

### QTL on Chromosome 2B

*QLr.usw-2BS* mapped to a chromosome region known to carry at least six designated leaf rust resistance genes: *Lr13* (Seyfarth et al., 2000), *Lr16* (McCartney et al., 2005), *Lr23* (McIntosh and Dyck, 1975), *Lr35* (Seyfarth et al., 1999), *Lr48* (Saini et al., 2002), and *Lr73* (Park et al., 2014). *Lr13*, *Lr35* and *Lr48* are reportedly APR genes, whereas Saragolla likely carries a major seedling resistance gene, based on the seedling evaluations conducted at CIMMYT. In addition, the molecular markers *Xbarc55* and *IWB35283*, previously reported to be linked to *Lr13* (Zhang et al., 2016), mapped at 72.1 cM and 74.2 cM, respectively, in the tetraploid wheat consensus map (Maccaferri et al., 2015), while the SNP markers *Tdurum_contig76118_145* and *wsnp_Ex_c18354_27181086* flanking *QLr.usw-2BS*, mapped at 8.4 cM and 12.3 cM, respectively, in the same consensus map. Also, *Lr35* is unlikely to be present in Saragolla since it was introgressed into hexaploid wheat from *Aegilops speltoides* and we did not detect molecular evidence that Saragolla is carrying this introgression. The SNP markers *IWB31002*, *IWB39832*, *IWB34324*, *IWB72894*, *IWB36920*, and *IWB70147* are reported to be co-segregating with the leaf rust resistance gene *Lr48* (Nsabiyera et al., 2016). However, none of these SNP markers are located within the genetic interval of *QLr.usw-2BS* in both the Saragolla/ATRED #2 genetic map (**Supplementary File S1**) and the tetraploid consensus map (Maccaferri et al., 2015). Therefore, it can be assumed that *QLr.usw-2BS* is different from *Lr48*. *Lr23* is also an unlikely candidate for *QLr.usw-2BS*, since the race BBG/BP used in this study is virulent to *Lr23* (Huerta-Espino et al., 2008; Huerta-Espino et al., 2011; Loladze et al., 2014). *Lr73* was identified in Australia in the common wheat genotype Morocco, which is widely susceptible to isolates of *P. triticina* (Park et al., 2014), including race BBBQJ with a virulence phenotype and SSR genotype similar to BBG/BP (Aoun et al., 2017). Hence, it is unlikely that *Lr73* is present in Sragolla. The all-stage resistance gene *Lr16* maps to the distal end of chromosome 2BS, and is closely linked to the SSR markers *Xwmc764*, *Xgwm210*, and *Xwmc661* (McCartney et al., 2005) and to KASP markers *kwm677* and *Kwm744* (Kassa et al., 2017). However, genotyping with these markers showed polymorphism between Saragolla and the *Lr16*-carrying AC Domain (**Supplementary Figures S5**, **S6**), which suggests that *QLr.usw-2BS* is different from *Lr16*. Aoun et al. (2017) identified *LrPI244061* that confers resistance to race BBBQJ of *P. triticina*, on chromosome 2BS of the durum landrace PI 244061. Based on the tetraploid wheat consensus map of chromosome 2B (Maccaferri et al., 2015), markers *IWB6117* and *IWB72183* linked to *LrPI244061* (Aoun et al., 2017) mapped at 42.8 cM and 45.8 cM, respectively, whereas markers *Tdurum_contig76118_145* and *wsnp_Ex_c18354_27181086*, flanking *QLr.usw-2BS*, mapped at 8.4 cM and 12.3 cM, respectively. In addition, marker *IWB72183* mapped at 133.8 cM on chromosome 2B_1, in the Saragolla/ATRED #2 genetic map (**Supplementary File S1**), while *QLr.usw-2BS* peaked at 82 cM. In summary, *QLr.usw-2BS* does not seem to include any of the six previously designated *Lr* genes and therefore is likely to harbor a previously uncharacterized gene.

### QTL on Chromosome 3B

Chromosome 3B is known to carry the race-specific resistance gene *Lr27* that requires the presence of the complementary gene *Lr31* on chromosome 4B, to confer leaf rust resistance in wheat (Mago et al., 2011). However, race BBG/BP emerged in 2008 in the state of Sonora, in northwestern Mexico, after acquiring virulence to the adult plant race-specific resistance gene *Lr12* and the seedling complementary resistance genes *Lr27*+*Lr31* (Huerta-Espino et al., 2008; Huerta-Espino et al., 2011). Therefore, *Lr27* is unlikely to be involved in the resistance to BBG/BP in Saragolla. The APR gene *Lr74* was identified in the hexaploid wheat population Ning7840 × Clark, and mapped to the short arm of chromosome 3B, closely linked to the SNP markers *IWA6651*, *IWA3724*, *IWA4654*, *IWA1702*, *IWA5203*, *IWA5202*, and *IWA5201* (Li et al., 2017). Kolmer et al. (2018b) identified a QTL for adult plant leaf rust resistance in the soft red winter wheat cultivar Caldwell, and mapped it very close to the *Lr74* locus. Based on the tetraploid wheat consensus map (Maccaferri et al., 2015), all of the SNP markers linked to *Lr74* mapped between positions 6 and 7.1 cM on chromosome 3BS, while markers *Tdurum_contig33168_461* and *RAC875_rep_c82061_78*, which flank *QLr.usw-3B* in Saragolla, mapped at 87 and 88 cM, respectively, in the same consensus map. In addition, marker *IWA3724* mapped at 15.9 cM on LG 3B_1 in the Saragolla/ATRED #2 map (**Supplementary File S1**), while *QLr.usw-3B* spanned the interval 11.5 to 13.5 cM on LG 3B_2. Therefore, we can assume that *QLr.usw-3B* is distinct from the APR gene *Lr74*. Recently, the new APR gene *Lr77* was mapped to chromosome 3BL in the hard red winter wheat cultivar “Santa Fe” (Kolmer et al., 2018c). The SNP markers *IWB32805* and *IWB10344* co-segregating with *Lr77*, mapped respectively at 148.4 cM and 151.6 cM in the tetraploid wheat consensus map, approximately 62 cM distal to the markers flanking *QLr.usw-3B* (Maccaferri et al., 2015). Furthermore, marker *IWB32805* mapped at 150.8 cM on LG 3B_2 in the Saragolla/ATRED #2 map, while *QLr.usw-3B* peaked at 13 cM on the same LG (**Supplementary File S1**). These large distances between the two intervals harboring *Lr77* and *QLr.usw-3B* indicate that they are two distinct *Lr* genes. *Lr79* is another newly mapped all-stage leaf rust resistance gene on chromosome 3BL, from the durum wheat landrace Aus26582 (Qureshi et al., 2018). Based on comparative analysis using the consensus 90K SNP genetic map (Wang et al., 2014) and the physical map of Chinese Spring (RefSeq v1.0), the authors estimated the distance between *Lr77* and *Lr79* at approximately 12 cM or 9.2 Mbp (Qureshi et al., 2018). Hence, it is possible to argue that *Lr79* does also map at a large genetic distance from *QLr.usw-3B*, and that the latter may be an uncharacterized leaf rust resistance gene.

### QTL on Chromosome 6B

Previous studies have reported three designated leaf rust resistance genes that map to the short arm of chromosome 6B: *Lr36* (Dvorak and Knott, 1990), *Lr53* (Marais et al., 2005), and *Lr61* (Herrera-Foessel et al., 2008a). Genes *Lr36* and *Lr53* were introgressed into chromosome 6BS of hexaploid wheat from *T. speltoides* and *T. dicoccoides*, respectively (Dvorak and Knott, 1990; Marais et al., 2005), but no reports of the transfer of either of these genes into durum wheat are available. Hence, these two genes are unlikely candidates for the major QTL *QLr.usw-6BS*, identified on the short arm of chromosome 6B in Gaza. However, since *T. dicoccoides* (genome AABB) is the wild progenitor of durum wheat, further genetic analysis and allelism tests would be required to fully rule out *Lr53* as a candidate. *Lr61* was identified in the CIMMYT-derived Chilean durum wheat cultivar Guayacan INIA, with linkage to marker *Xwmc487*, and is effective against the *P. triticina* race BBG/BP, predominant in northwestern Mexico (Herrera-Foessel et al., 2008a; Loladze et al., 2014). The presence of susceptible plants in the F_2_ progeny from the cross between Gaza and the *Lr61*-carrying Sooty_9/Rascon_37//Guayacan INIA indicated that *QLr.usw-6BS* was neither allelic nor linked to *Lr61* (**Supplementary Table S1**). Furthermore, Gaza showed polymorphism compared to the *Lr61* carrier Guayacan INIA, for the SSR marker *Xwmc487* (**Supplementary Figure S1**). Kthiri et al. (2018) identified two leaf rust resistance genes in the two durum cultivars Geromtel_3 and Tunsyr_2, conferring resistance to race BBG/BP of *P. triticina*, and mapping to the short arm of chromosome 6B. The authors showed that *Lr_Geromtel_3* and *Lr_Tunsyr_2* were either allelic or closely linked to each other and to *Lr61* (Kthiri et al., 2018; Kthiri, 2017). Allelism tests showed that the resistance gene in Gaza is either allelic or closely linked to the gene in Geromtel_3 but not to the gene in Tunsyr_2 (**Supplementary Table S1**). The Australian breeding line PI 209274 carries resistance to races BBBQJ and BBB/BN_*Lr61*vir of *P. triticina*, on chromosome 6BS (Aoun et al., 2017). Molecular markers *IWB39456* and *IWB416*, both linked to *LrPI209274* (Aoun et al., 2017), mapped at 5.6 cM in the Gaza/ATRED #2 linkage map (**Supplementary File S1**), close to the peak position of *QLr.usw-6BS* at 1.3 cM. Additional fine mapping studies and allelism tests are required to determine the relationship between *QLr.usw-6BS* and *LrPI209274*.

Several *Lr* genes have also been previously identified on the long arm of chromosome 6B. These include *Lr9*, which was translocated into hexaploid wheat from *Ae. umbellulata* (Schachermayr et al., 1994). However, there are no reports of this gene being transferred into durum wheat, thus, *Lr9* is unlikely a candidate gene for *QLr.usw-6BL*. The all-stage resistance gene *Lr3* is a known locus on the long arm of chromosome 6B with four reported alleles: *Lr3a*, *Lr3bg*, *Lr3ka*, and *Lr3d* (Kolmer, 2015c). Herrera-Foessel et al. (2007) identified and mapped *Lr3* and the closely linked gene *Lr_Camayo_* in the two durum wheat lines Storlom and Camayo, respectively. Although these two closely linked genes on chromosome 6BL confer resistance telo *P. triticina* races prevalent on durum wheat in Northwestern Mexico, allelism tests between Gaza and Cirno C2008, a carrier of *Lr_Camayo_*, suggested that the resistance to leaf rust in Gaza is unrelated to *Lr_Camayo_* (**Supplementary Table S1**). Furthermore, analysis of seedling and adult plant evaluation results confirmed the involvement of an APR gene for leaf rust resistance in Gaza, while both *Lr3* and *Lr_Camayo_* are all-stage resistance genes. Recently, Lan et al. (2017) identified *QLr.cim*-*6BL* that confers adult plant resistance to race BBG/BP of *P. triticina*, in the CIMMYT durum wheat line Bairds. Likewise, Aoun et al. (2017) mapped *LrPI387263* to the long arm of chromosome 6B, in the Ethiopian durum landrace PI 387263. Based on comparative mapping analysis, molecular markers linked to these genes mapped either very close to or overlapping the *QLr.usw-6BL* interval in the tetraploid wheat consensus map (Maccaferri et al., 2015) or the Gaza/ATRED #2 map (**Supplementary File S1**). Therefore, *QLr.usw-6BL*, *QLr.cim-6BL*, and *LrPI387263* could possibly harbor the same gene.

### QTL on Chromosome 7B

*QLr.usw-7BL* maps in a gene-dense region with several genes/QTL for resistance to rusts and other fungal diseases, including *Lr14a*, one of the most widely exploited *Lr* genes in wheat (Herrera-Foessel et al., 2008b; Terracciano et al., 2013), and the closely linked gene *Lr14b* (Dyck and Samborski, 1970), the slow-rusting APR gene *Lr68* (Herrera-Foessel et al., 2012), *LrBi16* which is allelic to *Lr14a* (Zhang et al., 2015), and *LrFun*, which is closely linked to *Lr14a* (Xing et al., 2014). The prevalent Mexican races of *P. triticina*, including race BBG/BP that is used in the present study, are virulent for *Lr14b* (Ordoñez and Kolmer, 2007; Herrera-Foessel et al., 2008b); therefore, it is unlikely that this gene is conferring leaf rust resistance in Arnacoris. *Lr68* is an adult-plant resistance gene that confers a slow-rusting phenotype, however, *QLr.usw-7BL* had a major effect on leaf rust resistance in Arnacoris, at both the seedling and adult plant stages (**Figure 3**), making *Lr68* an unlikely candidate for *QLr.usw-7BL*. When tested with the NBS-LRR primers *4406F/4840R*, both parental lines Arnacoris and ATRED #2 had the null allele (**Supplementary Figure S4**), indicating that *Lr14a* is not segregating in this population. Further investigation will be required to identify the specific gene responsible for the leaf rust resistance conferred by *QLr.usw-7BL*.

### Physical Mapping and Candidate Gene Identification

Anchoring of the SNP markers associated with the various QTL detected in the present study to the durum wheat reference genome identified several genes that encode for proteins known to be involved in plant pathogen interactions and disease resistance. So far, three race specific leaf rust resistance genes (*Lr1*, *Lr10*, and *Lr21*) have been cloned in wheat, and all three proteins contained NBS-LRR motifs (Feuillet et al., 2003; Huang et al., 2003; Cloutier et al., 2007). The APR gene *Lr34* protein is a full-size ABC transporter (Krattinger et al., 2009) while *Lr67* was shown to encode a recently evolved hexose transporter (Moore et al., 2015). Map-based cloning of *Yr36*, a gene that confers non-race-specific adult plant resistance to stripe rust in wild emmer wheat (*T. turgidum* ssp. *dicoccoides*), showed that it encodes a protein with a predicted kinase domain (Fu et al., 2009). The genes identified within the leaf rust resistance QTL intervals in Gaza, Arnacoris, and Saragolla have structures typical of disease resistance proteins and are excellent gene candidates for further studies.

## Conclusion

The present study identified six genomic regions involved in leaf rust resistance in durum wheat. The resistance to race BBG/BP of *P. triticina* in the Middle Eastern landrace Gaza was controlled by two QTL on chromosome 6B. *Qlr.usw-6BS* accounted for most of the phenotypic variance and is neither allelic nor linked to *Lr61*. The second QTL, *QLr.usw-6BL*, may be a new APR gene for leaf rust resistance in wheat. Likewise, the French cultivar Arnacoris carried two QTL for leaf rust resistance. *QLr.usw-1BL.1* mapped to the *Lr46* region; however, Arnacoris did not carry any of the *Lr46* molecular markers. The major QTL on chromosome 7B in Arnacoris, *QLr.usw-7BL*, explained most of the leaf rust phenotypic variance and was shown to be different from the widely deployed gene *Lr14a*. The Italian durum variety Saragolla carried a major QTL on chromosome 2B, designated as *QLr.usw-2BS*, which accounted for most of the phenotypic variance, as well as two minor QTL on chromosomes 3B and 1BL. Molecular marker analysis suggested that *QLr.usw-2BS* is distinct from *Lr16* while *QLr.usw-1BL.2* is likely the APR leaf rust resistance gene *Lr46*, and *QLr.usw-3B* is a potentially uncharacterized leaf rust resistance gene. Physical mapping of the SNP markers associated with these QTL to the durum wheat reference sequence enabled the identification of candidate genes for leaf rust resistance in these cultivars. With the availability of SNP markers tightly linked to all these QTL, some with major and other with minor effects, the durum wheat lines used in the present study can be used as donors to strategically combine genes with different modes of action to produce a more durable leaf rust resistance in durum wheat.

## Data Availability Statement

All datasets generated for this study are included in the manuscript/**Supplementary Files**.

## Author Contributions

CP and KA conceived and designed the study. KA developed the populations. AL produced the purified inoculum and conducted phenotyping and data collection. AL and DK analyzed the phenotypic data. SD performed DNA extraction. DK performed the genotyping and QTL analysis and drafted the manuscript. AN’D built the genetic maps. KN conducted the physical mapping. SW revised the manuscript. All authors contributed to manuscript revision, read, and approved the submitted version.

## Funding

We are grateful for the financial support of Genome Canada, Genome Prairie, Western Grains Research Foundation, Saskatchewan Wheat Development Commission, Alberta Wheat Development Commission, the Saskatchewan Ministry of Agriculture, Viterra, the Saskatchewan Innovation and Opportunity Scholarship, and the Rene Vandeveld Postgraduate Scholarship. CIMMYT’s contribution to the present work has been made possible thanks to funding from “Patronato para la Investigación y Experimentación Agrícola del Estado de Sonora (PIEAES),” “Fundacíón Produce Sonora, México,” and the Durable Rust Resistance Wheat (DRRW) project as part of the Borlaug Global Rust Initiative (BGRI).

## Conflict of Interest

The authors declare that the research was conducted in the absence of any commercial or financial relationships that could be construed as a potential conflict of interest.
