# Supplementary figures and images for: Mapping of Genetic Loci Conferring Resistance to Leaf Rust From Three Globally Resistant Durum Wheat Sources
Source: Front Plant Sci. 2019 Oct 8;10:1247. doi: 10.3389/fpls.2019.01247 (PMC6792298; doi:10.3389/fpls.2019.01247)

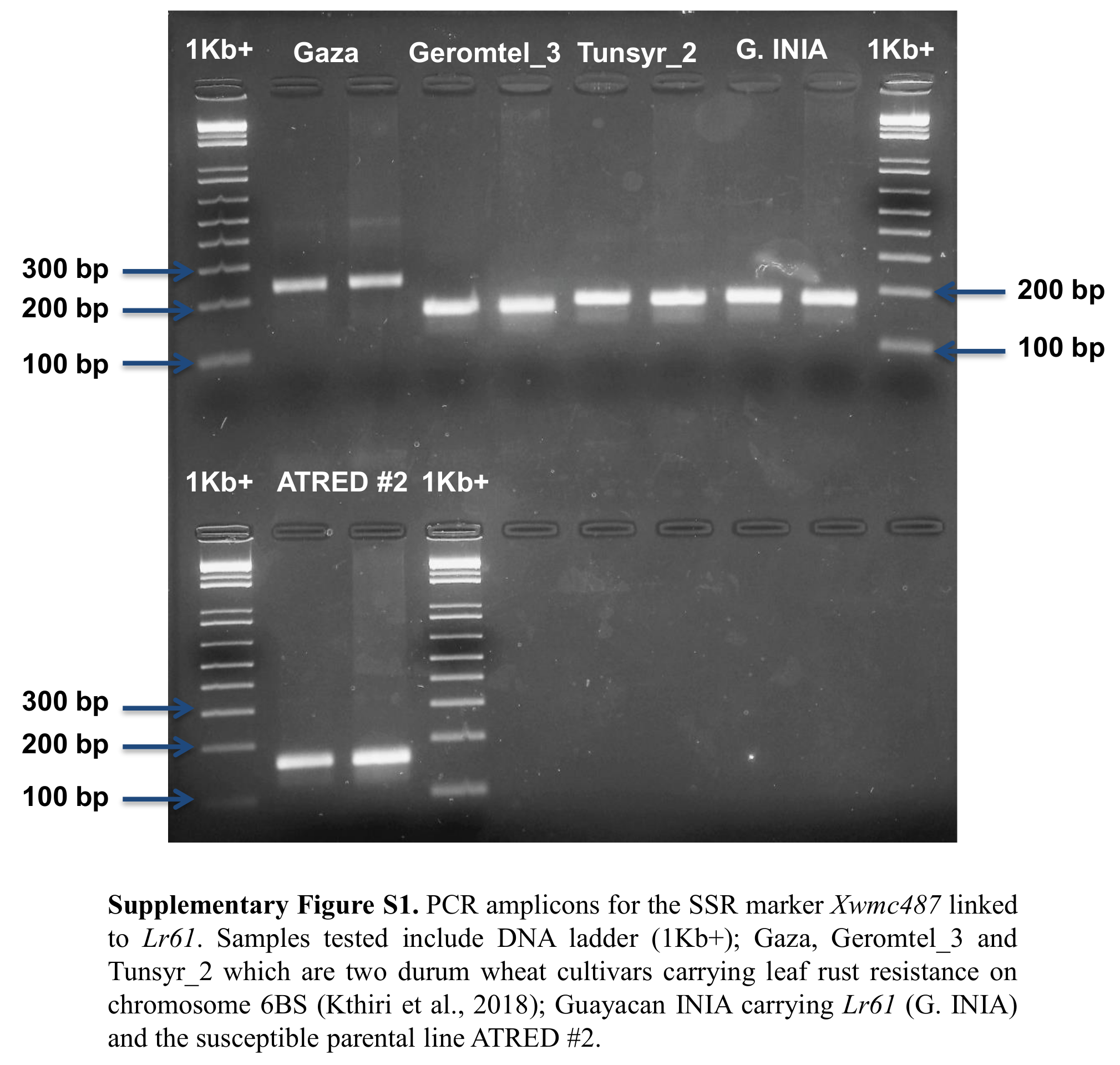

Supplement: Supplementary file 1 [file Image_1.tif]

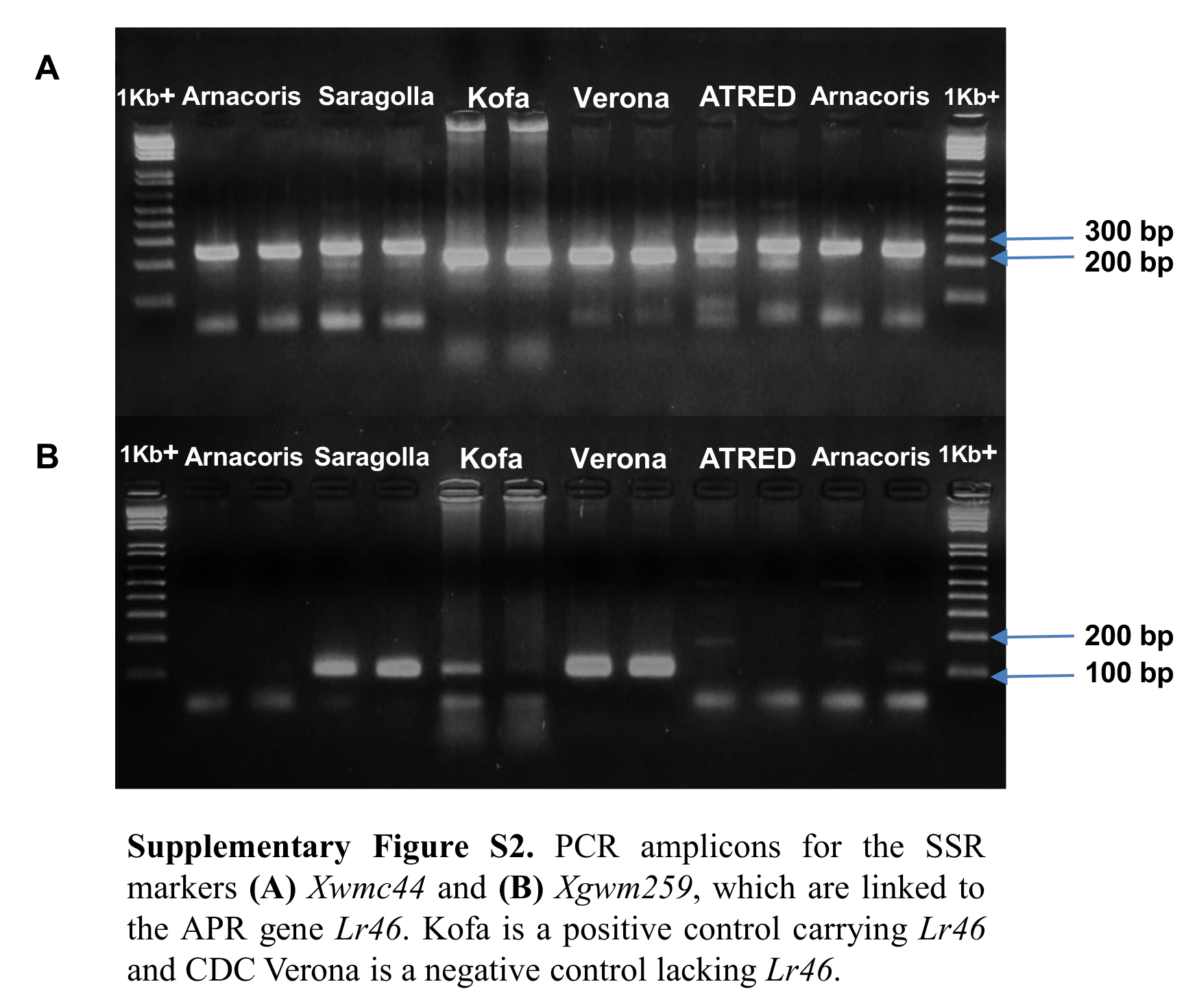

Supplement: Supplementary file 2 [file Image_2.tif]

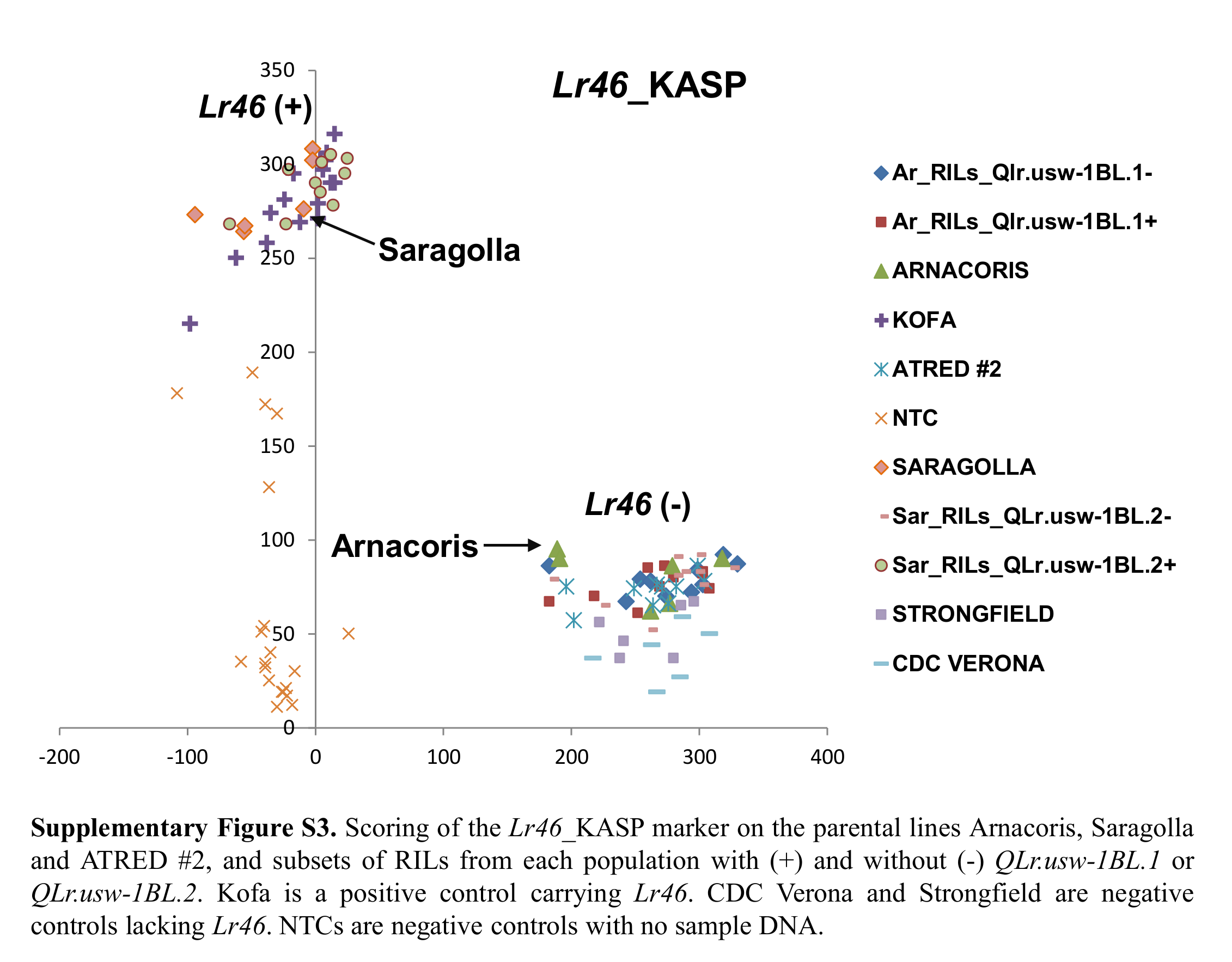

Supplement: Supplementary file 3 [file Image_3.tif]

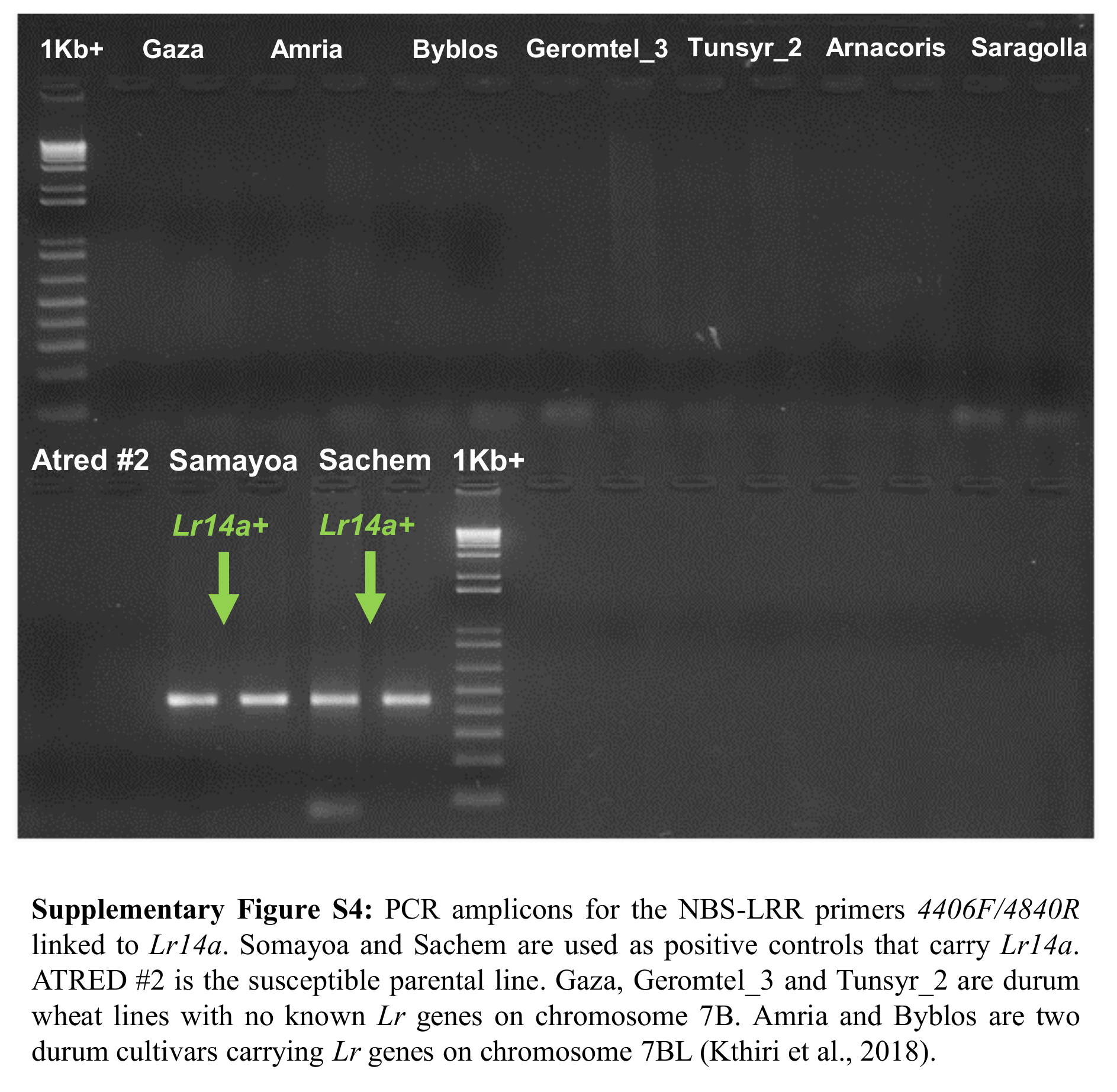

Supplement: Supplementary file 4 [file Image_4.tif]

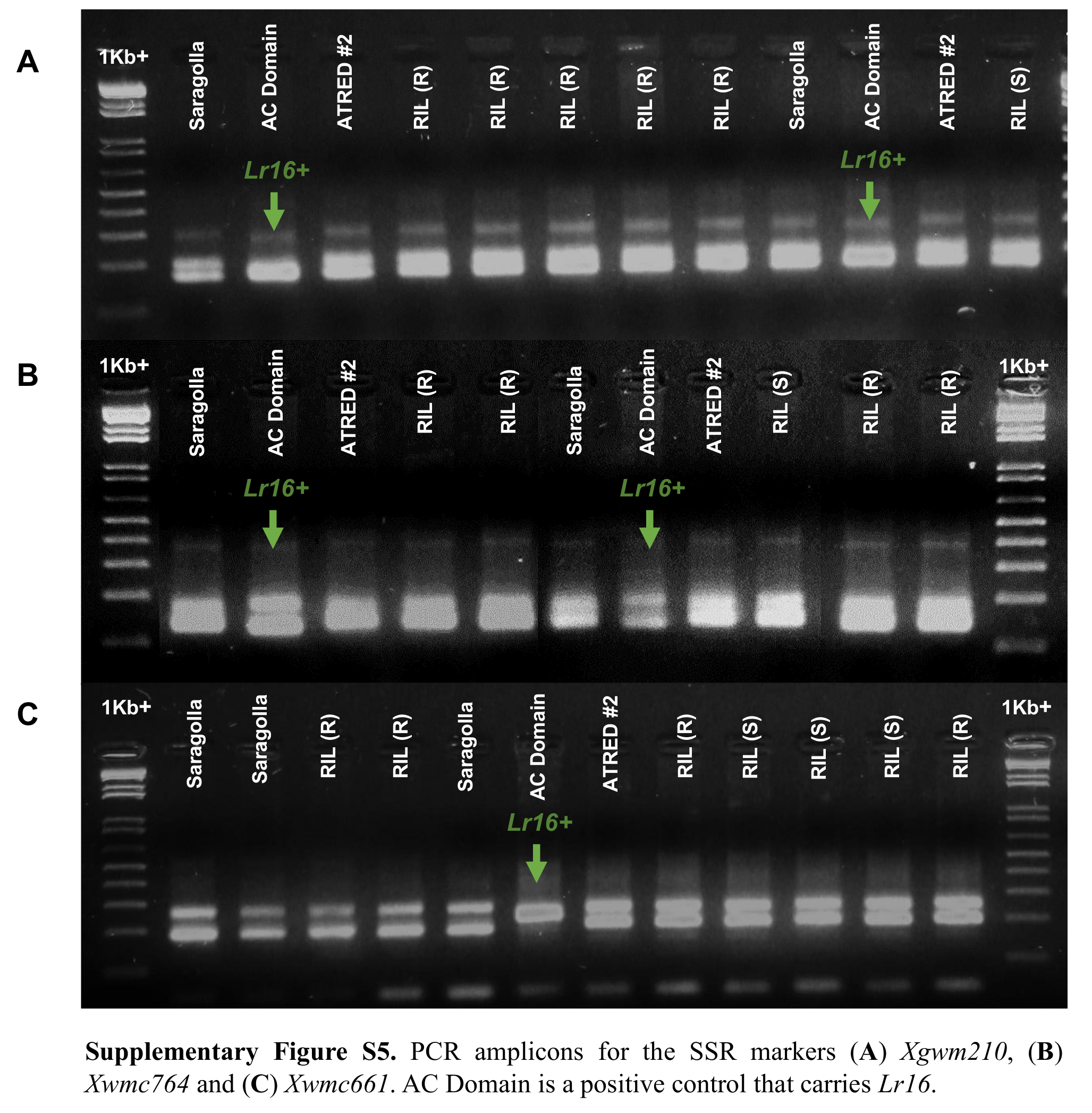

Supplement: Supplementary file 5 [file Image_5.tif]

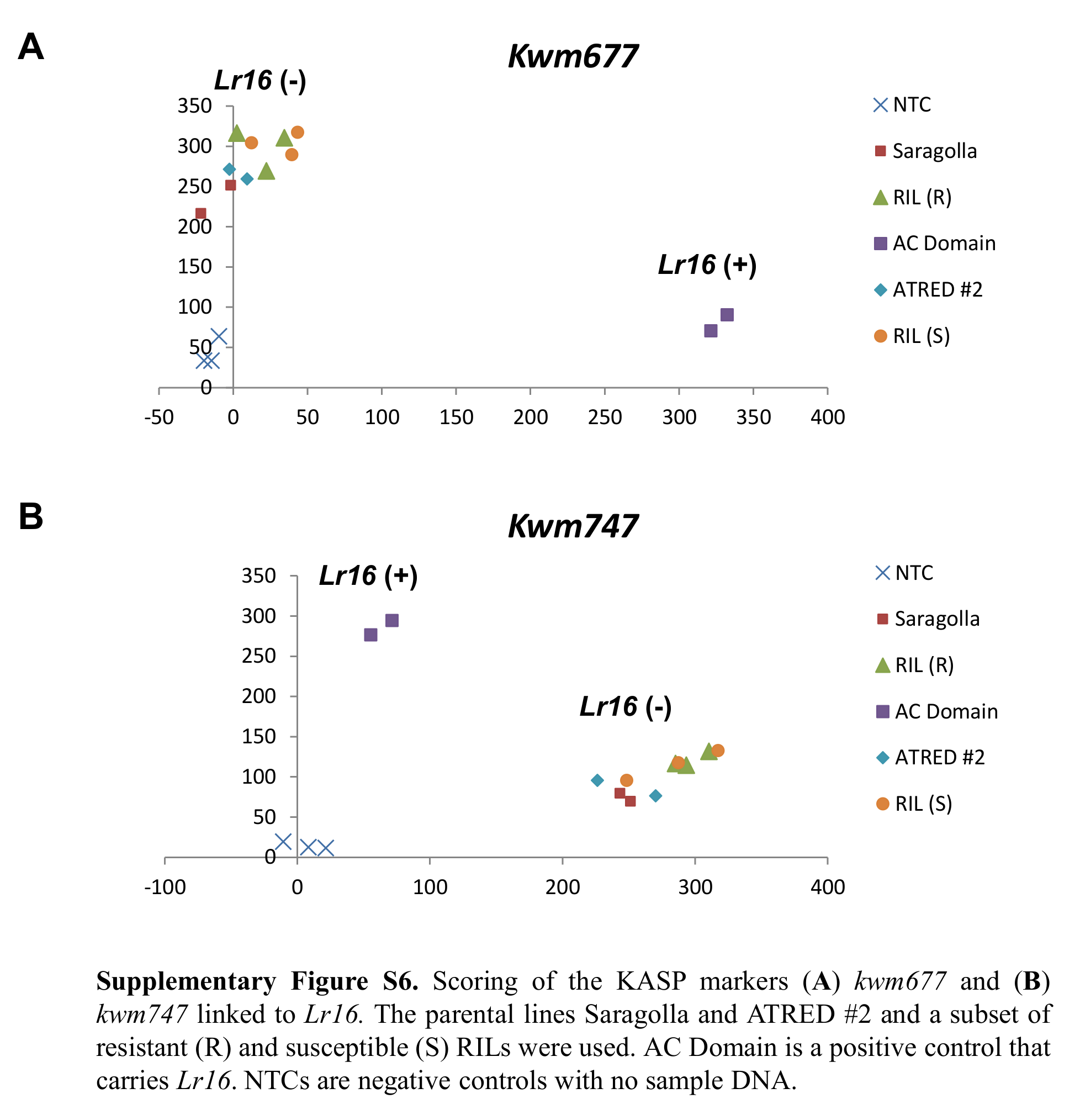

Supplement: Supplementary file 6 [file Image_6.tif]
