# Supplementary material for: Mapping of Genetic Loci Conferring Resistance to Leaf Rust From Three Globally Resistant Durum Wheat Sources
Source: Front Plant Sci. 2019 Oct 8;10:1247. doi: 10.3389/fpls.2019.01247 (PMC6792298; doi:10.3389/fpls.2019.01247)
Supplement: Supplementary file 7 [file Table_1.pdf]

Supplementary Table S1. Frequencies of resistant and susceptible F_2 plants for allelism testing involving Gaza and carriers of the leaf rust resistance genes *Lr61*, *Lr_Geromtel_3*, *Lr_Tunsyr_2* and Lr_{Camayo}.

**Cross**	**F_2 Progenies**					
	**Total**	**R**	**S**	**Ratio**	**χ^2**	***P***
Gaza/Sooty_9/Rascon_37/Guayacan INIA (*Lr61*)	177	153	24	55:9	0.007	0.93
Gaza/Geromtel_3	326	326	0	-	-	-
Gaza/Tunsyr_2	181	177	4	61:3	1.963	0.16
Gaza/Cirno C2008 (Lr_{Camayo})	273	257	16	61:3	0.599	0.44

The F_2 plants from each cross were categorized as resistant (R) or susceptible (S). Mendelian ratios and their corresponding *p-value* (*P*) from chi-square (χ^2) analysis are shown.
